# Ethical Issues of AI

**DOI:** 10.1007/978-3-030-69978-9_4

**Published:** 2021-03-18

**Authors:** Bernd Carsten Stahl

**Affiliations:** Centre for Computing and Social Responsibility, De Montfort University, Leicester, UK

**Keywords:** Ethical issues of AI, Empirical ethics, Ethics and machine learning, Ethics of digital society, Metaphysical issues

## Abstract

This chapter discusses the ethical issues that are raised by the development, deployment and use of AI. It starts with a review of the (ethical) benefits of AI and then presents the findings of the SHERPA project, which used case studies and a Delphi study to identify what people perceived to be ethical issues. These are discussed using the categorisation of AI technologies introduced earlier. Detailed accounts are given of ethical issues arising from machine learning, from artificial general intelligence and from broader socio-technical systems that incorporate AI.

Human flourishing as the foundation of AI ethics has provided the foundational basis for this book. We are now well equipped to explore ethical concerns in practice. This means that we now move from the conceptual to the empirical. In a first step I will give an overview of ethical issues, which I will then categorise in line with the earlier categorisation of concepts of AI.

## Ethical Benefits of AI

When we speak of ethical issues of AI, there tends to be an implicit assumption that we are speaking of morally bad things. And, of course, most of the AI debate revolves around such morally problematic outcomes that need to be addressed. However, it is worth highlighting that AI promises numerous benefits. As noted earlier, many AI policy documents focus on the economic benefits of AI that are expected to arise from higher levels of efficiency and productivity. These are ethical values insofar as they promise higher levels of wealth and wellbeing that will allow people to live better lives and can thus be conducive to or even necessary for human flourishing. It is worth pointing out that this implies certain levels of distribution of wealth and certain assumptions about the role of society and the state in redistributing wealth in ethically acceptable manners which should be made explicit. The EU’s High-Level Expert Group on AI (2019: 4) makes this very clear when it states:

AI is not an end in itself, but rather a promising means to increase human flourishing, thereby enhancing individual and societal well-being and the common good, as well as bringing progress and innovation.

AI offers several other technical capabilities that can have immediate ethical benefits. The International Risk Governance Center (2018) names AI’s analytical prowess, i.e. the ability to analyse quantities and sources of data that humans simply cannot process. AI can link data, find patterns and yield outcomes across domains and geographic boundaries. AI can be more consistent than humans, quickly adapt to changing inputs and free humans from tedious or repetitive tasks. These are all examples of technical capabilities that can easily be understood as being conducive to human flourishing because they lead to a better understanding and deeper insights into various phenomena. For instance, reducing commuting times or increasing the effectiveness of email spam filters are two everyday examples of where AI can make the life of busy professionals easier (Faggella 2020).

In addition to these examples of incidental ethical benefits, i.e. benefits that arise as a side effect of the technical capabilities of AI, there are increasing attempts to utilise AI specifically for ethical purposes. This is currently done under the heading of “ AI for Good” (Berendt 2019). The key challenge that AI for Good faces is to define what counts as (ethically) good. In a pluralistic world there may often not be much agreement on what is good or why it would be considered good. However, there have been numerous attempts (e.g. Holmes et al. 2011) to identify shared ethical goods or values, such as benevolence, security, achievement and self-direction.

One can observe two different approaches to identifying the ethical goods that AI would have to promote to count as AI for Good: substantive goods and procedures to achieve them. Substantive goods are those practical outcomes that are universally, or at least very broadly, accepted to be good. The dominant example of such substantive moral goods is the UN’s Sustainable Development Goals (SDGs) (Griggs et al. 2013). This set of 17 overarching goals has been described as “the world’s best plan to build a better world for people and our planet” (United Nations 2020). It arose from decades of discussion of development policy and sustainability and evolved from the UN’s Millenium Development Goals (Sachs 2012). The SDGs are interesting from an AI ethics perspective because they can be understood as the closest thing to humanity’s consensus in terms of moral aims. They have been adopted by the UN and most member states and now have a pervasive presence in ethical debates. In addition, they are not only aspirational, but broken down into targets and measured by indicators and reported on by the UN and member states annually. It is therefore not surprising that one of the most visible attempts to promote AI for Good by the UN’s International Telecommunications Union, the AI for Good Global Summit series,^1^ has the strapline “Accelerating the United Nations Sustainable Development Goals”.

While the SDGs are one dominant measure of the ethical benefit of AI, it is worth highlighting that they are not the only moral goods on which there is broad agreement. Another huge body of work that indicates broad global agreement is built around human rights (Latonero 2018). Just like the SDGs, these were developed by the UN and codified. In addition, human rights have in many cases become enforceable through national legislation and in local courts. Upholding human rights is a condition of human flourishing (Kleinig and Evans 2013)

SDGs and human rights are two ways of determining the ethical benefits of AI. They therefore play a crucial role in the discussion of how ethical benefits and issues can be balanced, as I will show in more detail below when we come to the discussion of how ethical issues can be addressed.

## Empirical Accounts of Ethical Issues of AI

There are numerous accounts of the ethical issues of AI, mostly developments of a long-standing tradition of discussing ethics and AI in the literature (Coeckelbergh 2019, Dignum 2019, Müller 2020), but increasingly also arising from a policy perspective (High-Level Expert Group on AI 2019). In this book and the SHERPA project^2^ that underpins much of the argument, the aim was to go beyond literature reviews and find out empirically what people have in mind when they speak of the ethical issues of AI. I will focus here on ten case studies and the open-ended first stage of a Delphi study to come to a better understanding of how the ethics of AI is perceived by people working with and on AI systems.

The level of analysis of the case studies was defined as organisations that make use of AI. Case studies are a methodology that is recommended to provide answers to the “how” and “why” of a phenomenon and events over which the researcher has little or no control (Yin 2003a, b). In order to gain a broad understanding, a set of application areas of AI was defined and case study organisations identified accordingly. Using this methodology, the case studies covered the following social domains:

- employee monitoring and administration
- government
- agriculture
- sustainable development
- science
- insurance
- energy and utilities
- communications, media and entertainment
- retail and wholesale trade
- manufacturing and natural resources

For each case a minimum of two organisational members were interviewed, the aim being to engage with at least one technical expert who understood the system and one respondent with managerial or organisational expertise. Overall, for the ten case studies, 42 individuals were interviewed. Based on the initial draft report of each case, peer review among the research team was undertaken, to ensure that the cases were consistent and comparable. For a detailed overview of the methods, findings and outcomes of the case study research, see Macnish et al. (2019)

The second piece of research that informs this chapter was the first stage of a three-stage Delphi study. Delphi studies are a well-established methodology to find solutions to complex and multi-faceted problems (Dalkey et al. 1969, Adler and Ziglio 1996, Linstone and Turoff 2002). They are typically expert-based and are used to find consensus among an expert population concerning a complex issue and to produce advice to decision-makers. Delphi studies normally involve several rounds of interaction, starting with broad and open questions, which are then narrowed down and prioritised.

The overview of ethical issues of AI that informs my discussion draws from the responses to the question in the first round of our Delphi Study. This was sent out to 250 experts on ethics and AI, selected from a range of stakeholders including technical experts, industry representatives, policymakers and civil society groups. Of these, 93 engaged with the online survey. A total of 41 usable responses were analysed. The open-ended question that was asked was: “What do you think are the three most important ethical or human rights issues raised by AI and/or big data?”

The analysis and findings of the first round were published and shared with the Delphi participants (Santiago 2020). These findings were then combined with the ones arrived at from the case study data analysis. Through group discussions similar relevant issues were combined and given suitable names or labels to ensure they were distinct and recognisable. For each of them a brief one-paragraph definition was provided.

The following list enumerates all the ethical issues that were identified from the case studies and the Delphi study, totalling 39.

1. Cost to innovation
2. Harm to physical integrity
3. Lack of access to public services
4. Lack of trust
5. “Awakening” of AI
6. Security problems
7. Lack of quality data
8. Disappearance of jobs
9. Power asymmetries
10. Negative impact on health
11. Problems of integrity
12. Lack of accuracy of data
13. Lack of privacy
14. Lack of transparency
15. Potential for military use
16. Lack of informed consent
17. Bias and discrimination
18. Unfairness
19. Unequal power relations
20. Misuse of personal data
21. Negative impact on justice system
22. Negative impact on democracy
23. Potential for criminal and malicious use
24. Loss of freedom and individual autonomy
25. Contested ownership of data
26. Reduction of human contact
27. Problems of control and use of data and systems
28. Lack of accuracy of predictive recommendations
29. Lack of accuracy of non-individual recommendations
30. Concentration of economic power
31. Violation of fundamental human rights in supply chain
32. Violation of fundamental human rights of end users
33. Unintended, unforeseeable adverse impacts
34. Prioritisation of the “wrong” problems
35. Negative impact on vulnerable groups
36. Lack of accountability and liability
37. Negative impact on environment
38. Loss of human decision-making
39. Lack of access to and freedom of information

There are several observations that could be made about this list. While in most cases one might intuitively accept that the issues can be seen as ethically relevant, no context or reason is provided as to why they are perceived to be ethically problematic. Many of them are not only ethically problematic but also directly linked to regulation and legislation. Being an ethical issue thus clearly does not exclude a given concern from being a legal issue at the same time.

The ethical issues are furthermore highly diverse in their specificity and likelihood of occurrence. Some are certain to come to pass, such as issues around data protection or data accuracy. Others are conceivable and likely, such as misuse or lack of trust. Yet others are somewhat diffuse, such as a negative impact on democracy, or on justice. In some cases, it is easy to see who should deal with the issues, while in others this is not so clear. This one-dimensional list of ethical issues is thus interesting as a first overview, but it needs to be processed further to be useful in considering how these issues can be addressed and what the priorities are.

**Fig. 4.1:**
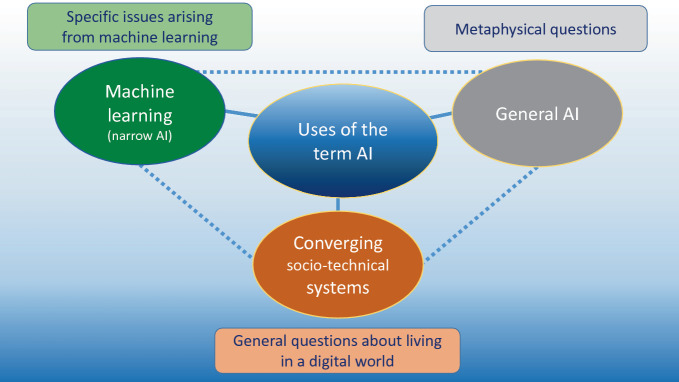
Concepts of AI and ethical questions they raise

It is possible to map the ethical issues to the different meanings of the concept of AI as outlined in Figure 10.1007/978-3-030-69978-9_2, as many of the issues are linked to the features of the different meanings as highlighted in Figure 10.1007/978-3-030-69978-9_2. I therefore distinguish three different sets of ethical issues: those arising from machine learning, general issues related to living in a digital world, and metaphysical issues (see Fig. 4.1).

Figure 4.1 indicates the relationship between the different categories of AI introduced in Chapter 10.1007/978-3-030-69978-9_2 and the ethical issues that will be discussed in the upcoming section. This relationship is indicative and should be understood as heuristic, i.e. problem-solving, as the relationships in practice are more complex and not necessarily as linear as the figure might suggest.

## Ethical Issues Arising from Machine Learning

The first set of issues consists of those that arise from the features of machine learning. Many of the machine learning techniques that led to the current success of AI are based on artificial neural networks. The features of these approaches that give rise to ethical concerns are opacity, unpredictability and the need for large datasets to train the technologies. Neither the developer, the deployer nor the user (see box) can normally know in advance how the system will react to a given set of inputs. And because the system learns and is thus adaptive and dynamic, past behaviours are not a perfect predictor for future behaviour in identical situations.

### Developer, Deployer and User

Most current AI policy work distinguishes between developers, deployers and users (European Parliament 2020). The developer is the technical expert (or organisation) who builds the system. The deployer is the one who decides its use and thus has control over risks and benefits. In the case of an autonomous vehicle, for example, the developer might be the car manufacturer, and the deployer might be an organisation offering mobility services. A user is the one benefiting from the services. These roles may coincide, and a developer may be a deployer. Making the distinction seems reasonable, however, because a developer can be expected to have detailed understanding of the underlying technology, whereas the deployer may have much less insight.

A primary and frequently cited ethical issue is that of privacy and data protection. Privacy and data protection are not identical (Buttarelli 2018), but for the purposes of AI ethics, the key privacy concern is informational privacy, and data protection can be understood as a means to safeguard informational privacy. AI based on machine learning poses several risks to data protection. On the one hand it needs large data sets for training purposes, and the access to those data sets can raise questions of data protection. More interesting, and more specific to AI, is the problem that AI and its ability to detect patterns may pose privacy risks, even where no direct access to personal data is possible. The classic study by Jernigan and Mistree (2009) claiming to be able to identify sexual orientation from Facebook friendships is a good example. Notwithstanding the ethical and scientific merits of this particular study, it is easy to see that AI can be used to generate insights that raise privacy concerns. AI also has the potential of allowing the re-identification of anonymised personal data in ways that were not foreseen before the capabilities of machine learning became apparent. While data protection law is well established in most jurisdictions, AI has the potential to create new data protection risks not envisaged by legislation and thereby create new ethical concerns. AI may also use or generate types of personal data currently less widely employed, such as emotional personal data, further exacerbating the situation (Tao et al. 2005, Flick 2016).

Data protection concerns are directly linked to questions of data security. Cybersecurity is a perennial problem of ICT, not just AI. However, AI systems may be subject to new types of security vulnerabilities, such as model poisoning attacks (Jagielski et al. 2018). Furthermore, these systems may be used for new types of vulnerability detection and exploitation (Krafft et al. 2020).

Privacy and data protection issues thus point to broader questions of reliability in AI systems. While reliability is a concern for all technical artefacts, the opacity of machine learning systems and their unpredictability mean that traditional deterministic testing regimes may not be applicable to them. The outputs of machine learning systems depend on the quality of the training data, which may be difficult to ascertain. The integrity of data can be threatened by security breaches, but also by technical or organisational aspects. This means that the reliability of machine learning systems may need to be assessed in different ways from other types of systems, which can be an ethical issue, if the system’s output affects ethical value. For example, an AI system used for the identification of disease markers in pathology may work well under research conditions, with well-labelled training data, and perform at the level of a trained pathologist, or even better, under such conditions. This does not guarantee that the same system using the same model would perform as well under clinical conditions, which may be one of the reasons why, despite the great promise that AI holds for medicine, there are relatively few AI systems already in clinical practice (Topol 2019).

Machine learning systems are by definition not transparent, or at least not transparent in the way that other ICT systems could be. Where they are proprietary systems, the commercial confidentiality of algorithms and models may further limit transparency. “Transparency” is itself a contested term, but lack of transparency raises questions of accountability (USACM 2017). Lack of transparency makes it more difficult to recognise and address questions of bias and discrimination.

Bias is a much-cited ethical concern related to AI (CDEI 2019). One key challenge is that machine learning systems can, intentionally or inadvertently, result in the reproduction of already existing biases. There are numerous high-profile accounts of such cases, for example when gender biases in recruitment are replicated through the use of machine learning or when racial biases are perpetuated through machine learning in probation processes (Raso et al. 2018). Discrimination on the basis of certain (sometimes so-called protected) characteristics is not just an ethical issue but has long been recognised as a human rights infringement, and such discrimination therefore tends to be illegal in many jurisdictions. As AI poses a risk to this human right, there has been a focus on highlighting the potential of machine learning to infringe the right to equality and non-discrimination (Access Now Policy Team 2018).

Safety is also a key ethical issue of machine learning, in particular in systems that interact directly with the physical world, such as autonomous vehicles (BmVI 2017) or systems governing critical healthcare provision. While currently not very visible in the public debate, safety is sure to emerge prominently when machine-learning-enabled systems start to physically engage with humans more broadly.

The ethical issues set out in this section are directly related to the technical characteristics of machine learning. There are, however, numerous other ethical concerns which are less clearly linked to machine learning, many of which have to do with the characteristics of broader socio-technical systems that are discussed in the next section.

## General Issues Related to Living in a Digital World

The second set of ethical issues consists of those that relate to what I called “AI as converging socio-technical systems”. In Section 2.3 I suggested that these systems have the characteristics of autonomy, social impact and manipulation. To be clear, the distinction is an analytical one, as the converging socio-technical systems are not separate from machine learning systems but tend to include these and be based on machine learning *and* other AI capabilities. The difference is more one of perspective, where the term “ machine learning” is used to focus on specific technologies for defined applications, whereas the converging socio-technical systems tend to involve numerous technologies and their focus is on the societal impact they cause.

I have chosen the label “living in a digital world” to describe these issues, in order to make it clear that most of them, while linked to AI, are not necessarily confined to AI. These questions are linked to broader societal and political decisions on how to structure and use large socio-technical systems. They can therefore not be viewed in separation from their societal role, and many of the ethical issues are directly caused by the way in which society and its actors work with these technologies.

An initial set of issues that arise from living in a digital world is related to the economy. The most prominent among these is likely to concern (un)employment. The potential of AI-related technologies to create a new wave of automation and thereby replace jobs has long been recognised (Collins 1990). In fact, Norbert Wiener suggested that computers competing with humans for jobs would have dire consequences for employment: “It is perfectly clear that this will produce an unemployment situation, in comparison with which the present recession and even the depression of the thirties will seem a pleasant joke” (Wiener 1954: 162).

While this bleak prediction has not (yet) come to pass, it is feared that AI will negatively affect employment. The novelty in the perceived threat from AI, which differs from earlier similar fears about ICT in general or other automation technologies, is that the jobs currently under apparent threat are better-paying ones: AI may increasingly imperil the income of middle-class professionals (Boden 2018). Losing employment is of course not only an economic problem; it also has social and psychological aspects (Kaplan and Haenlein 2019). The actual consequences of the introduction of AI for the employment market are at least partly an empirical question. The outcomes may be other than expected: jobs may not disappear but change instead (AI Now Institute 2017), and new jobs may be created, which may lead to new questions of fairness and distribution (House of Lords 2018).

The economic impacts of AI are not limited to employment. A further key concern is the concentration of economic (and by implication political) power. The reliance of current AI systems on large computing resources and massive amounts of data means that those organisations that own or have access to such resources are well placed to benefit from AI. The international concentration of such economic power among the big tech companies is independent of AI, but AI-related technologies have the potential to exacerbate the problem (Nemitz 2018).

These changes may not only be quantitative, i.e. related to the ability of large companies to make even more profits than they did prior to the use of AI, but may also be qualitatively different. Zuboff’s (2019) concept of “ surveillance capitalism” aims to capture the fundamental shifts in the economy that are facilitated by AI and the use of big data for behavioural prediction. Her argument is that these developments raise questions of fairness when large companies exploit user data that has been expropriated from individuals without compensation. The economic performance of large internet companies that make heavy use of AI certainly gives pause for thought. At the time of writing, Apple had just been valued as the most valuable global company, reaching a market value of $2 trillion. The stock market value of the five big internet companies – Apple, Microsoft, Amazon, Alphabet and Facebook – increased by $3 trillion during the COVID-19 pandemic, between 23 March and 19 August 2020 (Nicas 2020). This development may have more to do with the pathologies of the stock market than anything else, but it clearly shows that investors have huge hopes for the future of these companies – hopes that are likely to be related to their ability to harness AI.

Notwithstanding these astonishing figures, probably an even more important problem is that such companies utilise their insights to structure the space of action of individuals, thereby reducing the average citizen’s ability to make autonomous choices. Such economic issues are thus directly related to broader questions of justice and fairness. There are immediate questions, such as the ownership of data and how this translates into the possibility of making use of the benefits of new technologies. Intellectual property has been a hotly debated topic in the ethics of computing for a long time (Spinello and Tavani 2004) and is now spilling over into the AI debate.

Another hotly debated issue is that of access to justice in the legal sense and how AI will transform the justice system. The use of AI for predictive policing or criminal probation services can broaden existing biases and further disadvantage parts of the population (Richardson et al. 2019).

While the use of AI in the criminal justice system may be the most hotly debated issue, AI is also likely to have impacts on access to other services, thereby potentially further excluding segments of the population that are already excluded. AI can thus exacerbate another well-established ethical concern of ICT, namely the so-called digital divide(s) (McSorley 2003, Parayil 2005, Busch 2011). Well-established categories of digital divides, such as the divides between countries, genders and ages, and between rural and urban, can all be exacerbated due to AI and the benefits it can create. These benefits imply that a lack of ability to access the underlying technology leads to missed opportunities, which can be an ethical concern.

Another basic category of ethical issues in the digital world is that of freedom. It is easy to see how the freedom of an individual whose parole decision was made or influenced by AI would be affected. However, the influence of AI on freedom is broader and more subtle. By providing or withdrawing access to information the technologies that surround us shape the space of possible action. The argument goes beyond Lessig’s (1999) point that ICT is a form of law that allows or disallows certain actions. ICT in general and AI in particular can make a human’s options appear or disappear without that human being aware of it. This does not even have to imply a conscious desire to mislead or deceive, but is simply an expression of the fact that our social reality is technically mediated and this mediation has consequences. An example would be the results of an internet search engine. Search engines rely heavily on AI. They also structure what users can see and will thus perceive as relevant, and how they then act. Search engine providers use this as part of their business model, by displaying paid-for content more prominently and enticing users to purchase. The point is, however, that even without such conscious attempts to direct users’ attention, a search engine would still structure users’ perception of reality and thus their scope of action.

As in the other cases, this is not necessarily negative. AI can open up enormous opportunities and create spaces for actions that were previously unthinkable, for example by allowing partially sighted people to drive vehicles autonomously, or by creating personalised medical solutions beyond what is currently possible. But at the same time, it can reduce individual autonomy, removing the freedom to decide and act in more or less subtle ways. An example might be the use of AI to steer visitors to a city on routes that avoid congestion and promote the safety of tourists (Ryan and Gregory 2019). Such a system is based on morally desirable aims, but it still reduces the ability of individuals to move about the city as they would do in the absence of the system. This does not have to be an ethical issue, but it may have unintended consequences that are ethically problematic, for example when it reduces the footfall in parts of the city that depend on visitors.

Broader societal issues are power relationships and power asymmetries. Economic dominance and the structuring of options for action may give large amounts of power and dominance to some actors, to the point where democratic principles are jeopardised. The scandal around Facebook and Cambridge Analytica (Isaak and Hanna 2018) is a high-profile reminder of the potential vulnerabilities of democratic processes. But, as Coeckelbergh (2020: 100) points out, it is not just a problem of new forms of surveillance, manipulation and authoritarianism. Our democratic structures may be similarly undermined by “changing the economy in a way that turns us all into smartphone cattle milked for our data”, thus linking back to Zuboff’s pervasive theme of surveillance capitalism.

The list of possibly problematic issues of AI in different application areas is as long as the list of possible benefits. In most of these areas there are difficult questions about how to identify benefits and costs and what to do about them. A high-profile example is the use of AI for the creation of autonomous weapons. While it is easy to see that saving soldiers’ lives by replacing them with robots would be an ethical benefit, there are numerous counterarguments ranging from the practical, such as the reliability of such systems, to the political, such as whether they would lower the threshold to starting wars, to the fundamental, such as whether it can ever by appropriate to take human lives on the basis of machine input (Sharkey 2017, Defense Innovation Board 2019, Babuta et al. 2020).

Similar worries arise in AI for health, where technology can improve diagnoses and treatments, but may have risks and downsides. An example would be care technologies: robotic systems have long been proposed as a way of addressing challenges faced by the care sector, but there are concerns about replacing human contact with technology, which is often seen as a fundamental ethical issue (Decker 2008, Sharkey and Sharkey 2010, Goeldner et al. 2015).

These broader societal issues are not confined to direct impact on human lives and actions, but also take in the impact of AI on the environment. While AI offers the possibility of decreased power consumption by streamlining processes, it simultaneously requires large amounts of resources and it creates new products and services that can have negative impacts on the environment.

## Metaphysical Issues

This discussion of ethical issues of AI started with the most immediate issues arising from a specific technology, namely machine learning, and then progressed to broader societal concerns. The third and final category of ethical issues, what I call “metaphysical issues”, is the most open and unexplored one. I have used the term “metaphysical” because the issues here are directly linked to fundamental aspects of reality, of the nature of being and human ability to make sense of this. They also go to the heart of the nature of humans and humanity.

These metaphysical issues are mostly related to artificial general intelligence (AGI) or good old-fashioned AI (GOFAI), which is typically conceptualised in terms of a symbolic and logical representation of the world. The idea is that AGI (which may build on GOFAI, but does not have to) would display human reasoning abilities. To reiterate a point made earlier: there currently are no AGI systems available, and there is considerable disagreement about their possibility and likelihood. I am personally not convinced that they are possible with current technologies, but I cannot prove the point any more definitively than others, so I will remain agnostic on the point of fundamental possibility. What seems abundantly clear, however, is that progress in the direction of AGI is exceedingly slow. Hence, I do not expect any technology that would be accepted as AGI by the majority of the expert communities to come into existence during the coming decades.

The metaphysical ethical issues raised by AGI are therefore not particularly urgent, and they do not drive policy considerations in the way that issues like discrimination or unemployment do. Most policy documents on AI ignore these issues, on the implicit assumption that they are not in need of policy development. In the empirical research presented earlier in this section, these metaphysical issues were not identified as issues that organisations currently engage with. There is probably also an element of fear on the part of scholars and experts of being stigmatised as not being serious or scholarly, as these metaphysical issues are the staple of science fiction.

I nevertheless include them in this discussion of ethical issues of AI for two reasons. Firstly, these questions are thought-provoking, not only for experts but for media and society at large, because they touch on many of the fundamental questions of ethics and humanity. Secondly, some of these issues can shed light on the practical issues of current AI by forcing clearer reflection on key concepts, such as autonomy and responsibility and the role of technology in a good society.

The techno-optimistic version of AGI is that there will be a point when AI is sufficiently advanced to start to self-improve, and an explosion of intelligence – the singularity (Kurzweil 2006) – will occur due to a positive feedback loop of AI onto itself. This will lead to the establishment of super-intelligence (Bostrom 2016). The implication is that AGI will then not only be better than humans at most or all cognitive tasks, but will also develop consciousness and self-awareness (Torrance 2012). The contributors to this discussion disagree on what would happen next. The super-intelligent AGI might be benevolent and make human life better, it might see humans as competitors and destroy us, or it might reside in a different sphere of consciousness, ignoring humanity for the most part.

Speculations along those lines are not particularly enlightening: they say more about the worldview of the speculator than anything else. But what is interesting is to look at some of the resulting ethical issues in light of current technologies. One key question is whether such AGIs could be subjects of responsibility. Could we hold them morally responsible for their actions or the consequences of these actions (Bechtel 1985)? To put it differently, is there such a thing as artificial morality (Wallach and Allen 2008, Wallach et al. 2011)? This question is interesting because it translates into the question: can we hold current AIs responsible? And this is a practical question in cases where AIs can create morally relevant consequences, as is the case for autonomous vehicles and many other systems that interact with the world.

The question whether an entity can be a subject of moral responsibility, i.e. someone or something of which or whom we can say, “X is responsible,” hinges on the definition of responsibility (Fischer 1999). There is a large literature on this question, and responsibility subjects typically have to fulfil a number of requirements, which include an understanding of the situation, a causal role in events, the freedom to think and act, and the power to act, to give four examples.

The question of whether computers can be responsible is therefore somewhat similar to the question of whether they can think. One could argue that, if they can think, they can be responsible. However, Turing (1950) held the question of whether machines can think to be meaningless and proposed the imitation game, i.e. the Turing test, instead. In light of the difficulty of the question it is therefore not surprising that an analogous approach to machine responsibility was devised, the moral Turing test, where the moral status of a machine could be defined by the fact that it was recognised as a moral agent by an independent interlocutor. The problem with that approach is that it does not really address the issue. I have elsewhere suggested that a machine that can pass the Turing test could probably also pass a moral Turing test (Stahl 2004).

Much of the discussion of the moral status of AI hinges on the definition of “ ethics”. If one takes a utilitarian position, for example, it would seem plausible to assume that computers would be at least as good as humans at undertaking a moral calculus, provided they had the data to comprehensively describe possible states of the world. This seems to be the reason why the trolley problem is so prominent in the discussion of the ethics of autonomous vehicles (Wolkenstein 2018). The trolley problem,^3^ which is based on the premise that an agent has to make a dilemmatic decision between two alternatives, either of which will typically kill different actors, has caught the attention of some scholars because it seems to map to possible real-world scenarios in AI, notably with regard to the programming or behaviour of autonomous vehicles. An autonomous vehicle can conceivably be put in a situation that is similar to the trolley problem in that it has to make a rapid decision between two ethically problematic outcomes. However, I would argue that this is based on a misunderstanding of the trolley problem, which was devised by Philippa Foot (1978) as an analytical tool to show the limitations of moral reasoning, in particular utilitarianism. The dilemma structure is geared towards showing that there is not one “ethically correct” response. It has therefore been argued (Etzioni and Etzioni 2017), rightly in my opinion, that the trolley problem does not help us determine whether machines can be ethical, because it can fully be resolved with recourse to existing standards of human responsibility.

I have argued earlier that the key to understanding ethics is an understanding of the human condition. We develop and use ethics because we are corporeal, and hence vulnerable and mortal, beings who can feel empathy with others who have fears and hopes similar to our own. This is the basis of our social nature and hence of our ethics. If we use this starting point, then AI, in order to be morally responsible and an ethical agent, would have to share these characteristics. At the moment no system comes close to empathy. This has nothing to do with AI’s computational abilities, which far exceed ours and have done for some time, but arises from the fact that AI is simply not in the same category as we are.

This does not mean that we cannot assign a moral status to AI, or to some type of AI. Humans can assign such a status to non-humans and have always done so, for example by viewing parts of nature or artefacts as divine or by protecting certain entities from being treated in certain ways.

Such a view of AI has the advantage of resolving some of the metaphysical questions immediately. If an existentialist commitment to our shared social world is a condition of being an ethical agent, then current AI simply falls out of the equation. This does not mean that developers of autonomous vehicles do not need to worry any more, but it does mean that they can use established mechanisms of responsibility, accountability and liability to make design decisions. It also does not fundamentally rule out artificial moral agents, but these would have to be of a very different nature from current computing technologies.

This position does not solve all metaphysical questions. There are interesting issues arising from the combination of humans and machines that need attention. Actor-networks containing AI-enabled artefacts may well change some of our ethical perceptions. The more AI gets integrated into our nature, the more it raises new questions. This starts with seemingly trivial aspects of the prevalence of ubiquitous devices such as mobile phones and what these do to our agency. Cutting-edge technologies, such as AI-supported brain computer interfaces, change what we can do, but they can also change how we ascribe responsibility. In this sense questions of posthumanism (Barad 2003) and human enhancement (Bostrom and Sandberg 2009, Coeckelbergh 2011) may be more interesting from the AI ethics perspective because they start with existing ethical agency that may need to be adjusted.

Much more could of course be said about ethical issues of AI, but this chapter has hopefully given a good overview and provided a useful categorisation of these issues, as shown in Table 4.1.

**Table 4.1:**
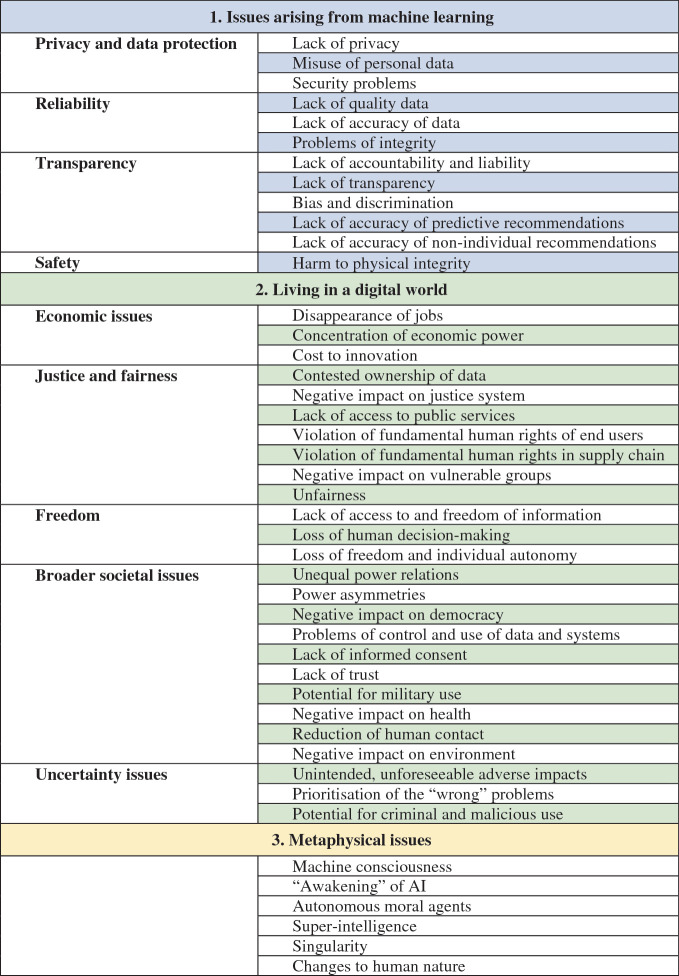
Three categories of ethical issues of artificial intelligence

The categorisation in Table 4.1 is not authoritative, and others are possible. A different view that would come to similar conclusions would focus on the temporal nature of the issues. Ordering ethical issues of AI by temporal proximity and urgency is not new. Baum (2018) has suggested the distinction between “presentists” and “futurists”, calling attention to near-term and long-term AI issues. Extending this thought to the discussion of ethical issues of AI as presented in this chapter, one can say that the ethical issues of machine learning are the most immediate ones and the metaphysical ones are long-term, if not perpetual, questions. The category of issues arising from living in the digital world is located somewhere between. This view may also have implications for the question of how, when and by whom ethical issues in AI can be addressed, which will be discussed in the next chapter.

## Footnotes

1 https://aiforgood.itu.int/.

2 https://www.project-sherpa.eu/

3 A typical trolley problem would see an agent standing near the points where two railway lines merge into a single track. From the single track, a train approaches. Unaware of the train, a number of children are playing on the left-hand track, whereas a single labourer, also unaware of the train, is working on the right-hand track. The train is set to hit the children. By switching the points, the agent can switch the train onto the right-hand track, thereby saving the children’s lives, but leading to a single death. What should the agent do? That is the trolley problem.
